# Supplementary material for: MetaRibo-Seq measures translation in microbiomes
Source: Nat Commun. 2020 Jun 29;11:3268. doi: 10.1038/s41467-020-17081-z (PMC7324362; doi:10.1038/s41467-020-17081-z)
Supplement: Supplementary file 10 — Supplementary Data 7 [file 41467_2020_17081_MOESM10_ESM.zip › File2/Confidence_VeryHigh_Taxonomy/323482_out.krona.html]

Javascript must be enabled to view this page.

members
magnitude
magnitudeUnassigned
count
unassigned
taxon
rank

323482\_out

8

superkingdom
2
8

8
1239
phylum

8
186801
class

order
186802
8

186806
family
8

8
genus
1730

1

SRS077502\_contig\_number\_contig-100\_154.85406
1262879
species

1

SRS148159\_contig\_number\_41678
1263078
species

2

SRS143342\_contig\_number\_11740SRS148721\_contig\_number\_39590
species
165185

species
39488

SRS017521\_contig\_number\_31674SRS019068\_contig\_number\_70340SRS057478\_contig\_number\_5159SRS144183\_contig\_number\_18730
4
